# An evaluation study on phenotypical methods and real-time PCR for detection of *Mycobacterium tuberculosis* in sputa of two health centers in Iran

**Published:** 2017-02

**Authors:** Shirin Sadr, Davood Darban-Sarokhalil, Gholam Reza Irajian, Abbas Ali Imani Fooladi, Jaleh Moradi, Mohammad Mehdi Feizabadi

**Affiliations:** 1 Department of Microbiology, School of Medicine, Tehran University of Medical Sciences, Tehran, Iran; 2 Department of Microbiology, School of Medicine, Iran University of Medical Sciences, Tehran, Iran; 3 Applied Microbiology Research Center, Baqiyatallah University of Medical Sciences, Tehran, Iran; 4 Pediatric Infectious Diseases Research Center, Tehran University of Medical Sciences, Tehran, Iran

**Keywords:** *Mycobacterium tuberculosis*, Real-time PCR, Cytochrome P450 cyp141

## Abstract

**Background and Objectives:**

For further confirmation of the previous in-house real-time PCR and CYP 141 target as a rapid and cheap diagnostic technique and a new target for direct detection of *Mycobacterium tuberculosis*, we evaluated and compared the results of smear, culture and real-time PCR in sputa that were collected from 2 health centers. Moreover we tried to evaluate the diagnostic accuracy of phenotypical methods for detection of tuberculosis that have been applied in two health centers of Iran.

**Materials and Methods:**

Thirty two sputa (including 15 smear positive and 17 smear negative) and 53 Sputa (29 smear and culture positive and 24 smear and culture negative specimens) were collected from tuberculosis suspected patients from health center No. 1 and 2 respectively. A Taqman probe was used for direct detection of *M. tuberculosis* using the specific primers.

**Results:**

Because of the results, data of health center No. 1 was not reliable. The average number of bacteria that was detected in health center No. 2 by real time PCR was 1.2E+003–7.3+004; 1.4E+004–1.29+005 and 8.27E+005–1.07+006 for one to three plus smear result groups, respectively. The overall sensitivity, specificity, positive predictive value (PPV) and negative predictive value (NPV) of real-time PCR were 96.5% (28/29), 95.8% (23/24), (96.6%) and (96%), respectively.

**Conclusion:**

Compared with the results of previous studies and being a good correlation between real-time PCR and phenotypic methods, emphasize that CYP141 is a good target for quantification of *M. tuberculosis* in sputa and real-time PCR can be a good method for evaluation of smear microscopy. Moreover, further surveillance is needed to evaluate the phenotypical methods and final decisions that are taken in health centers of Iran that can be observed and evaluated by the cheap molecular methods like in-house methods.

## INTRODUCTION

According to the world Health Organization (WHO) report, the incidence of Tuberculosis (TB) (per 100000 people) in Iran was 13.00 in 2015 and this disease is one of the most important infectious disease that kills approximately 2 million people in the world annually (1, 2).

Developing more drug resistance in *M. tuberculosis* and recent spreading of nontuberculous mycobacteria (NTM) in patients have forced the microbiologist to improve the rapidity, sensitivity and specificity of diagnostic tests (1, 3). There are several targets that have been used for molecular detection of *M. tuberculosis* specially IS6110 and 16S rDNA (4–8). One of those targets was Cytochrome P450 cyp141 that has been interoduced and used recently (9–11). The specificity and sensitivity of the target was checked for different species of mycobacteria and potentially pathogenic bacteria in the respiratory tract (9). The target was found specific for *M. tuberculosis* complex (*M. tuberculosis, M. bovis, M. bovis BCG, M. africanum* and *M. microti*) (9).

Despite of advantages of real-time PCR’s (high sensitivity, specificity, speed, simplicity and safety), further studies on the clinical samples are needed to prove real-time PCR as sensitive as culture for detection of tuberculosis (12). Moreover for more evaluation of new target (CYP 141) and more comparison of conventional methods with real-time PCR for quantitative detection of *M. tuberculosis* in clinical specimens, we decided to use the new target for detection of tuberculosis in clinical specimens of 2 health centers.

According to the previous published and unpublished studies (9, 10), the validity of the smear microscopy in some regions of Iran was lower than other regions. The definitive diagnosis is still made according on the smear microscopy results in most of the regions of Iran, especially in suburb ones. So the misdiagnosis leads to treat some non-TB cases and miss some TB patients. Based on the mentioned reasons, the other purpose in our study was to evaluate the smear microscopy results and final decisions that are made in health centers of Iran by real-time PCR.

## MATERIALS AND METHODS

### Specimen collection.

Sputa (n=85) were collected from the Iranian tuberculosis-suspected patients from 2 health centers in different cities from February 2013 to July 2014. Thirty two sputa (including 15 smear positive and 17 smear negative) and 53 sputa (29 smear and culture positive and 24 smear and culture negative specimens) were collected from suspected tuberculosis patients from health center No. 1 and 2, respectively. Patients were not under anti tuberculosis therapy before collecting the specimens. Direct smears were done in health centers according to the Ziehl-Neelsen staining method and Petroff methods. Culture was done in helath centers independently. Moreover the smears and culture results were read and interpreted according to WHO guidelines (13, 14).

### DNA extraction, real-time PCR analysis and quantification.

The previous probe and primers (Cyp-P 5′-Fam-ACAGCATGGCTCGTCACTCGC-BHQ-1-3′) (Tm = 65.0) and the primers (Cyp-F 5′-ATGACAAGCACCTCGATTCC-3′, Cyp-R 5′-TCGGACAGCACTCCCTTTAC-3′) (10), were used in real-time PCR. Preparation of standards was done according to the previous study (10). Standards were run nine times (triplicate assays on three separate days) before including them in each run of amplification. *M. tuberculosis* H37Rv strain and water were used as positive and negative controls respectively. Kappa test with p-value of 95% confidence was used by SPSS version 13.0

The optimized reaction was exactly like the previous study including 0.2 mM dNTP, 1× reaction buffer (50 mM KCl, 10 mM Tris-HCl [pH 9.0], 1.5 mM MgCl_2_), 2U of Taq DNA-polymerase (mi Taq,), 0.2 mM of each primer, 0.1 mM of probe (Metabion, Martinsried, Germany) and 10 μl of template DNA in total volume of 45 μl. The program was initial denaturation at 94°C for 10 min followed by 40 cycles of 94°C for 25s, 58°C for 55s.

Like previous studies the extraction of DNA from clinical specimens was done by commercial DNA extraction kit (Invitek, Germany). Finally, 10 μl of collected liquid was used for real-time PCR. DNA from all clinical specimens were extracted and subjected to real time PCR in double and the mean number of target copies was recorded.

## RESULTS

### Phenotypical and real-time PCR results of health centers.

After doing real-time PCR on clinical samples of health center No. 1, in comparison to the results of smear and culture, the results of real-time PCR were disappointing and there were not good co-relation between them. Moreover the results of smear microscopy and culture that were done in the health center were not matched (data are not shown). There were some specimens with smear positive but culture negative results. Of 17 smear and culture negative specimens, 4 were positive in terms of real-time PCR and real-time PCR did not detected *M. tuberculosis* in 9 specimens (out of 15 smear and culture positive). As mentioned above, since the results and specimens of health center No. 1 were not reliable, so we did not include these specimens for getting sensitivity and specificity of the target.

The results of smear microscopy and culture of health center No. 2 was reliable and was matched with the results of real-time PCR. In compare with the results of culture, the overall sensitivity, specificity, positive predictive value (PPV) and negative predictive value (NPV) of real-time PCR were 96.5% (28/29), 95.8% (23/24), (96.6%) and (96%), respectively. The average number of bacteria that was detected in health center No. 2 by real time PCR was 1.2E+003–7.3+004; 1.4E+004–1.29+005 and 8.27E+005–1.07+006 for one to three plus smear result groups, respectively.

There was not amplification with human white blood cell’s DNA, while amplification was found with Beta actin, in each reaction. The results of real time PCR and quantification of samples of health center No. 1 comparing to MTB result name and Ct value range of Xpert MTB/RIF (Cepheid) are shown in Table 1. Furthermore we compared the results of our study with previous study (Table 2) that was done on samples from other regions of Iran.

**Table 1.** The results of smear and culture reading and Real-time PCR (mean) in health center No.1comparing to MTB result name and Ct value range of Xpert MTB/RIF (Cepheid)

**No. of samples**	**Smear**	**Culture**	**Real-time PCR (Mean)**	**MTB result**	**Ct range**
Group 1: 20	+	+/++	1.2E+003 to 7.3+004	Low	22–28
Grooup 2: 7	++	++/+++	1.4E+004 to1.29+005	Medium	16–22
Group 3: 2	+++	+++/Innumerable	8.27E+005 to1.07+006	High	<16

**Table 2.** Comparison of the results of real-time PCR (mean) of this study with the results of previous study

**No. of samples (This study)**	**Smear**	**Real-time PCR (Mean) (This study)**	**No. of samples (Previous study)**	**Real-Time PCR (Previous study)**
**20**	+	1.2E+03 to 7.3+04	**23**	5.5E+04 to 8.5E+03
**7**	++	1.4E+04 to1.29+05	**48**	1.1E+06 to 7.2+04
**2**	+++	8.27E+05 to1.07+06	**17**	8.1E+07 to 1.2+06

## DISCUSSION

Like previous studies (9, 10), treatment process, DNA extraction protocol and quality of Taq DNA-polymerase were the main factors that affected on the results of real-time PCR.

The co-relation between real-time PCR and phenotypical results in specimens of health center No. 2 was almost like previous study (Table 1 and 2) (10). Despite of a little difference between the real-time PCR results of the studies, there was good correlation between them. As shown in Table 2, the results of real-time PCR in specimens with a smear value of 1-plus in both studies are similar, but the results of real-time PCR in specimens with a smear value of 2 and 3-plus are different. The main reason for this issue is probably due to the different numbers of samples that used in the studies. The number of 1-plus value specimens in both studies is almost similar, but the numbers of 2 and 3-plus specimens are different. Since the numbers of the specimens in the previous study are more, so the results of that study are more reliable.

Like previous study the correlation between phenotypical and real-time PCR is almost similar to the results of the Xpert MTB/RIF kit that published by Cepheid Inc (Table 1) (15). According to the kit, the Ct value <16 shows the high number of the DNA copy that means in our study more than 1×10^6^ copies/ml that showed in specimens with smear 3 plus codes. The optimized real time PCR in this study also could detect as little as 100 copies of the target which is similar to the previous study (sensitivity of culture:100 bacilli /mL^−1^) (12).

The conventional methods (smear microscopy and culture) for detection of *M. tuberculosis* are easy and cheap, and they are used in the world specially in developing countries. Using culture for all the suspected-tuberculosis specimens in most of the third-world countries like Iran is not possible and smear microscopy is still the main laboratory method for diagnosis of tuberculosis and the final decision and definitive diagnosis is made on the results of smear microscopy (16, 17). The sensitivity of smear microscopy is modest and variable (50%–80%) depending upon identified unexpected procedural deficiencies, the type of specimen, patient population, stain used and the experience of the microscopist. Despite the lower sensitivity and specificity of smear microscopy than culture and molecular methods, some studies disclosed if this method did correctly; it would detect most of tuberculosis disease (16–18).

The results of the specimens in health center No. 1 were completely different and showed a lot of false negative and positive results in comparison to the molecular method. As mentioned above even the results of smear and culture were not matched. The main reasons for the misdiagnosis by phenotypical methods are contamination of slides during the staining, not using appropriate protocols for treatment and diagnosis, contamination by non-tuberculosis mycobacteria and technical errors. Since using commercial molecular methods are too expensive specially for developing countries, a correct in-house molecular method can use for evaluation of phenotypical methods in the future observational studies in health centers and can be excellent alternative for rapid and correct detection of tuberculosis.
